# Complications Associated with Insertion of Intrauterine Pressure Catheters: An Unusual Case of Uterine Hypertonicity and Uterine Perforation Resulting in Fetal Distress after Insertion of an Intrauterine Pressure Catheter

**DOI:** 10.1155/2012/517461

**Published:** 2012-08-13

**Authors:** Kara M. Rood

**Affiliations:** Bridgeport Hospital, Yale New Haven Health System, Bridgeport, CT 06610, USA

## Abstract

Insertion of intrauterine pressure catheters is a routine procedure performed in labor and delivery departments, with few associated complications. There are several reports of maternal and neonatal morbidity associated with the use of intrauterine pressure catheters and their rare adverse outcomes. We report an unusual case of uterine hypertonicity resulting in fetal distress, immediately after the placement of an intrauterine pressure catheter. An emergent Cesarean section was performed for fetal distress and revealed a 5 cm vertical rent in the posterior lower uterine segment. The uterine perforation was repaired intraoperatively. Mother and infant did well and were discharged home on postoperative day four.

## 1. Introduction

Intrauterine pressure catheters (IUPCs) are used during labor to measure the frequency, duration, and strength of uterine contractions. It has been suggested that IUPCs are more accurate than external tocodynamometry, in detecting strength and frequency of uterine contractions, especially in cases of maternal obesity and/or suspected labor dystocias [1]. It is estimated that IUPCs are used in approximately 15% of deliveries preformed in the United States [2].

Uterine perforation is a rare reported complication, associated with the placement of intrauterine pressure catheters. This may be due to its rarity, but most likely due to its underreporting. Proper technique of placing intrauterine pressure catheters and being aware of the potential complications can lead to an early diagnosis and appropriate intervention.

## 2. Case Report

A 21-year-old Hispanic, nulliparous woman at 41.4 wks gestation with a body mass index (BMI) of 45 presented to labor and delivery department for scheduled induction of labor for postdates. Uneventful pregnancy. Initial cervical exam revealed favorable bishop score of 9. Induction was started with pitocin. Due to patient's elevated BMI, the external tocodynamometry was unable to detect all of the patient's reported contractions. Patient progressed to a dilatation of 4 cm and received effective epidural anesthesia. Despite augmentation with pitocin, amniotomy, and palpable uterine contractions, patient was arrested at 4 cm dilation for 3 hours.

Due to the difficultly identifying contractions, placement of an IUPC was performed to better characterize the frequency and strength of the uterine contractions. The IUPC was placed into the posterior aspect of the uterus under sterile technique, on the first attempt and with careful attention to ensure that the external catheter did not extend past the examiner's fingers. The IUPC was advanced to 45 cm with a small amount of resistance and no amniotic fluid was observed in the catheter. Immediately after placement, fetal bradycardia occurred for approximately 7 minutes, with fetal heart rate into the 60's. During the fetal bradycardia, routine fetal intrapartum resuscitation was preformed, IUPC was removed with no evidence of blood clot or vaginal bleeding, and palpation of uterus revealed uterine hypertonicity.

At this point, patient was consented for emergent cesarean section for fetal bradycardia. A live male infant was delivered from occiput anterior position, weight of 7.8 lbs and APGARS 9/9, at 1 and 5 minutes, respectively. Cord gases were within normal limits. Placenta was removed manually and inspected with no evidence of placental abruption. Uterus was cleared of all clots and debris, bowel was then identified within the uterine cavity. Inspection of the posterior aspect to the lower uterine segment revealed a 5 cm vertical rent in the lower uterine segment, medial to the insertion of the right uterosacral ligament. The uterus was then exteriorized and a 3 cm posterior hematoma was identified, medial to the uterine perforation.

A uterine perforation was identified and repaired in two layers, using 0 dexon and 2–0 chromic suture. Adequate hemostasis was noted and the posterior hematoma remained stable. Infant and mother were transferred to recovery in stable condition. Mother was informed of the uterine perforation associated with placement of the intrauterine pressure catheter and that she was not a candidate for future trial of labor after cesarean section. Review of anatomy ultrasound at 20 weeks of gestation showed a posterior placenta.

## 3. Discussion

 The placement of intrauterine pressure catheters is viewed as routine procedures on labor and delivery suites, to provide reliable and quantifiable measure of contraction duration, frequency, and strength. The strength of uterine contractions are expressed in Montevideo units (MVUs). Montevideo units measure the uterine pressure above baseline tone and are calculated by multiplying the number of uterine contractions over a ten-minute period. Research has shown that MVUs exceeding 200 are adequate for the active phase of labor [3].

 As to date, the American College of Obstetricians and Gynecologists do not recommend routine use of IUPCs. They suggest that IUPCs may be helpful in situations where external methods do not provide a clear tracing of contractions, such as, in cases of maternal obesity or when response to oxytocin is limited [1]. Euliano et al. showed that external tocodynamometry may be unreliable in obese patients and that contraction detection correlated better with intrauterine pressure catheters than with external tocodynamometry [4]. IUPCs may also be helpful to determine adequate contractions by measuring Montevideo units in suspected diagnosis of arrested labor. Several studies have shown that IUPCs provide more precise contraction monitoring, although the overall maternal and neonatal outcomes such as rate of operative delivery and adverse neonatal outcomes have shown no difference [5, 6].

 With the increasing number of obese women in the United States, the prevalence of maternal obesity (defined as prepregnancy body mass index [BMI] ≥30 kg/m^2^) has increased over a 10-year period, from 13% to 22% [7]. Maternal obesity poses several obstacles for the obstetrician during labor; including difficultly monitoring uterine contractions. This obstacle may contribute to an increasing role for IUPCs, in monitoring uterine contractions during labor augmentation in pregnancies complicated by maternal obesity. Therefore, it is important to be aware of the potential complications associated with placement of IUPCs.

 Extramembranous placement of IUPCs, between the uterine wall and the fetal membranes, have been reported to occur in approximately 14–38% of IUPC placements [8]. Fortunately, extra-membranous placement is usually associated with very few complications [9]. Uterine perforation is a rare complication quoted to be between 1 in 300 and 1 in 1400 in the literature, usually due to improper placement; in the extramembranous space [10]. The insertion of IUPCs have also been associated with other adverse outcomes, such as placental abruption, placental vessel perforation, cord entanglement, endometritis, and anaphylactoid syndrome [8–14].

 Several cases reports have described complications associated with extra-membranous placement of IUPCs [8–11]. Lind describes a hypertonic uterus immediately after extramembranous placement of IUPC, resulting in fetal bradycardia. Removal of the IUPC revealed a 6 inch blood clot in lumen tip of the IUPC, corresponding with intraoperative placenta inspection of a marginal placental abruption [10]. Madanes et al. report a case and reviewed five cases of uterine perforation after IUPC placement, as a result of inserting the rigid external cannula beyond the fingers of the examiner. Two of the six cases of uterine perforation, describe uterine hypertonicity, which resulted in fetal distress requiring an emergent cesarean section [9].

 Review of the literature suggests that uterine perforations are often asymptomatic and therefore underreported. Uterine perforation should be suspected when there is no uterine recorded waveform in the presence of a soft, nontender contracting uterus. Whereas, evidence of vaginal bleeding and/or uterine hypertonicity after placement of IUPC should raise suspicion for a possible placental abruption [15]. Proper technique when inserting an IUPC may help to minimize some of the adverse outcomes. For example, a sterile technique should be maintained; if any resistance is felt, it is important to stop advancing the catheter, avoid placing the rigid introducer past the examiner's fingers, visualize amniotic fluid in catheter immediately after placement, and insert IUPC away from placental site.

 This case report demonstrates the importance of knowing the location of placenta before inserting an IUPC. It is speculated that the IUPC was inserted extramembranous, due to absence of amniotic fluid present in the clear catheter and the resistance felt during insertion was due to the posteriorly located placenta. The uterine perforated then occurred in the thin, labored lower uterine segment just inferior to the placental attachment site. It is recommended that the catheter should be inserted posteriorly or anteriorly, opposite of known placental site, in order to minimize complications [3]. Inserting the catheter laterally may increase risk of placental vessel perforation; an unusual but possibly fatal complication [12].

 This is the third reported case of an IUPC insertion resulting in uterine hypertonicity and fetal distress immediately after uterine perforation in the literature; however, it is unknown how many cases have occurred and gone unreported. With the limitations of external tocodynamometry associated with maternal obesity and the rise in the number of obese pregnant women, the use and reported complications of IUPCs are expected to rise. Reviewing proper placement technique may help avoid mishaps, and bring awareness to the possible complications. This can lead to earlier recognition and appropriate intervention of the unusual complications associated with inserting an IUPC.
